# Indocyanine green fluorescence imaging localization-assisted thoracoscopy revision surgery after repair of esophageal atresia

**DOI:** 10.1186/s12876-022-02444-1

**Published:** 2022-08-05

**Authors:** Yanan Zhang, Murong Wang, Shuangshuang Li, Junmin Liao, Kaiyun Hua, Shen Yang, Jinshi Huang

**Affiliations:** Department of Neonatal Surgery, Beijing Children’s Hospital, Capital Medical University, National Center for Children’s Health, Beijing, 100045 China

**Keywords:** Recurrent tracheoesophageal fistula (rTEF), Fluorescence imaging thoracoscopy, Indocyanine Green (ICG), Revision surgery, Esophageal atresia

## Abstract

Revision surgery for the complications after repair of esophageal atresia is often complex because of previous surgeries and chest infections and thus requires surgical expertise. This study describes surgical experiences with the use of indocyanine green (ICG) fluorescence imaging localization-assisted thoracoscopy during revision surgery, including recurrent tracheoesophageal fistula (rTEF) (8 cases, one of which was esophageal-pulmonary fistula) and delayed esophageal closure (1 case). We performed fistula repair and esophageal reconstruction according to the indications of ICG. The application of this method avoids the excessive trauma caused by freeing the trachea and esophagus. Contrast imaging taken one week and one month after surgery indicated no spillover of the contrast agent from the esophagus, except in 1 case. Indocyanine green fluorescence imaging localization-assisted thoracoscopy is worth promoting for revision surgery after esophageal atresia repair.

## Background

The repair of an esophageal atresia is usually followed by a good outcome, however, the repair may fail or become complicated resulting in morbidity. Reoperation after the repair of esophageal atresia is often complex and requires experienced surgeons. The common condition requiring surgical repair include recurrent tracheoesophageal fistula (rTEF), which occurs in up to 5–14% of patients [1, 2], and delayed primary closure. The reoperations of rTEF are mainly divided into endoscopic and operative repairs, and surgical treatment remains dominant [3–5]. In recent years, thoracoscopic surgery for the repair of rTEF has been increasingly reported. Reoperations can be challenging because severe adhesion, scar tissue, and inflammation are usually found around the fistula, which is difficult even for experienced surgeons to locate. We investigated a better method for intraoperatively locating the surgical site.

Indocyanine green (ICG) is a medical dye that is safe and has many potential applications. ICG has been widely used for liver and heart function tests and other procedures, as well as for pediatric surgery [6, 7]. In recent years, ICG fluorescence imaging has also been used in laparoscopic surgeries, such as repeated nephrectomy, lymph tracking, and others, to improve intraoperative visualization of anatomical structures [8]. It has not been reported in revision surgery for complications after the repair of esophageal atresia. Based on our extensive experience with thoracoscopic repair surgery, we explored revision surgery for complications after the repair of esophageal atresia with ICG fluorescence imaging technique thoracoscopy.

## Materials and methods

### Patient selection

We performed a single-institution retrospective analysis of 8 patients undergoing repair for rTEF (1 esophageal-pulmonary fistula) and one case of delayed closure of the esophageal over a 4-month period (July 2021 and December 2021); in all cases, the ICG fluorescence imaging system was utilized. Institutional review board approval was obtained for this study. The patients listed in Table 1 required reoperation twice or three times. Patient demographics were collected and included age, sex, weight, diagnosis and comorbidities. Bronchoscopy and esophageal contrast imaging are routinely performed for diagnosis. ICG skin tests (0.1 ml, 0.125 mg/ml, subcutaneously) were done in all patients to avoid the allergy to iodides. All of them were negative.

**Table 1:** The 9 patients characteristics

Age (m, min–max)	10 (3–24)
Weight (kg)	7.55 (4.3–10.5)
Sex (n: male; female)	Male 8; Female 1
Number of operations	2.4 (2, 3)
rTET	8 (1 LEF)
Second-stage EA	1
Classification of primary EA	Gross-IIIA1/B8
*Comorbidities*	
Inguinal hernia	1
Horseshoe kidney	1
Congenital heart disease	2
Rib deformity	1
Hiatal hernia	1
Gastroesophageal reflux	3
Tracheomalacia	4
Tracheal stenosis	5
Anorectal malformation	1
Hydronephrosis	1
Vertebral deformity	2
Pyloric stenosis	1

### Surgical procedure

#### rTEF operation

##### rTEF in the main trachea

**Fig. 1:**
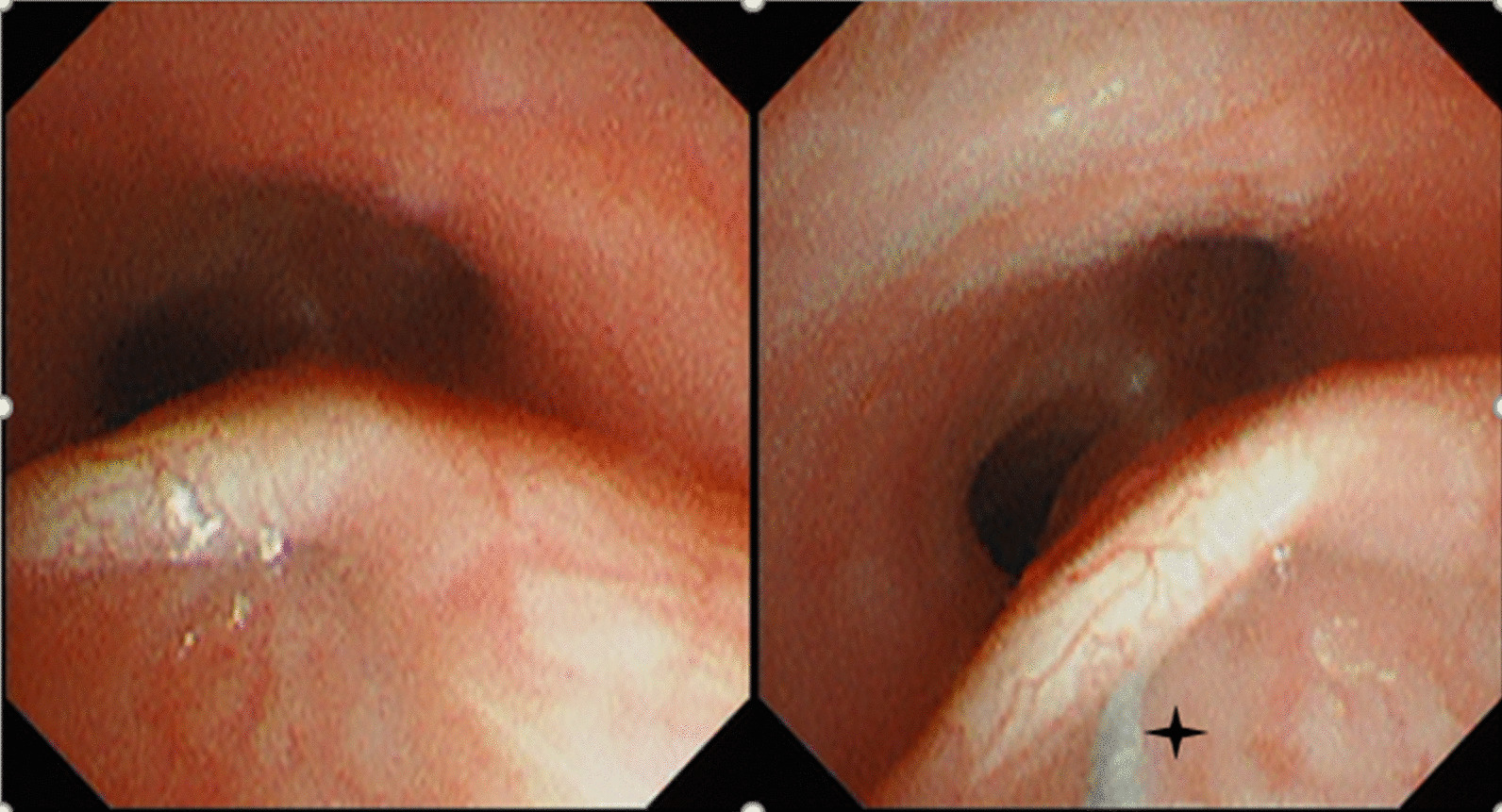
rTEF under the bronchoscopy, black four pointed star for a guide wire through the fistulae

0.625 mgICG (0.5 ml, concentration 1.25 mg/ml) diluted with sterile water was sprayed into the fistulae under bronchoscopy surveillance. A guide wire was simultaneously placed from the trachea to the esophagus and received from the esophagus via gastroscopy (Fig. 1). A thread was tied on the end of the guide wire, and the guide wire was replaced with the thread. Then, both ends of the thread were fixed at the corner of the mouth. In two cases, intubation with the guide wire could not be performed because of complex fistulae.

**Fig. 2:**
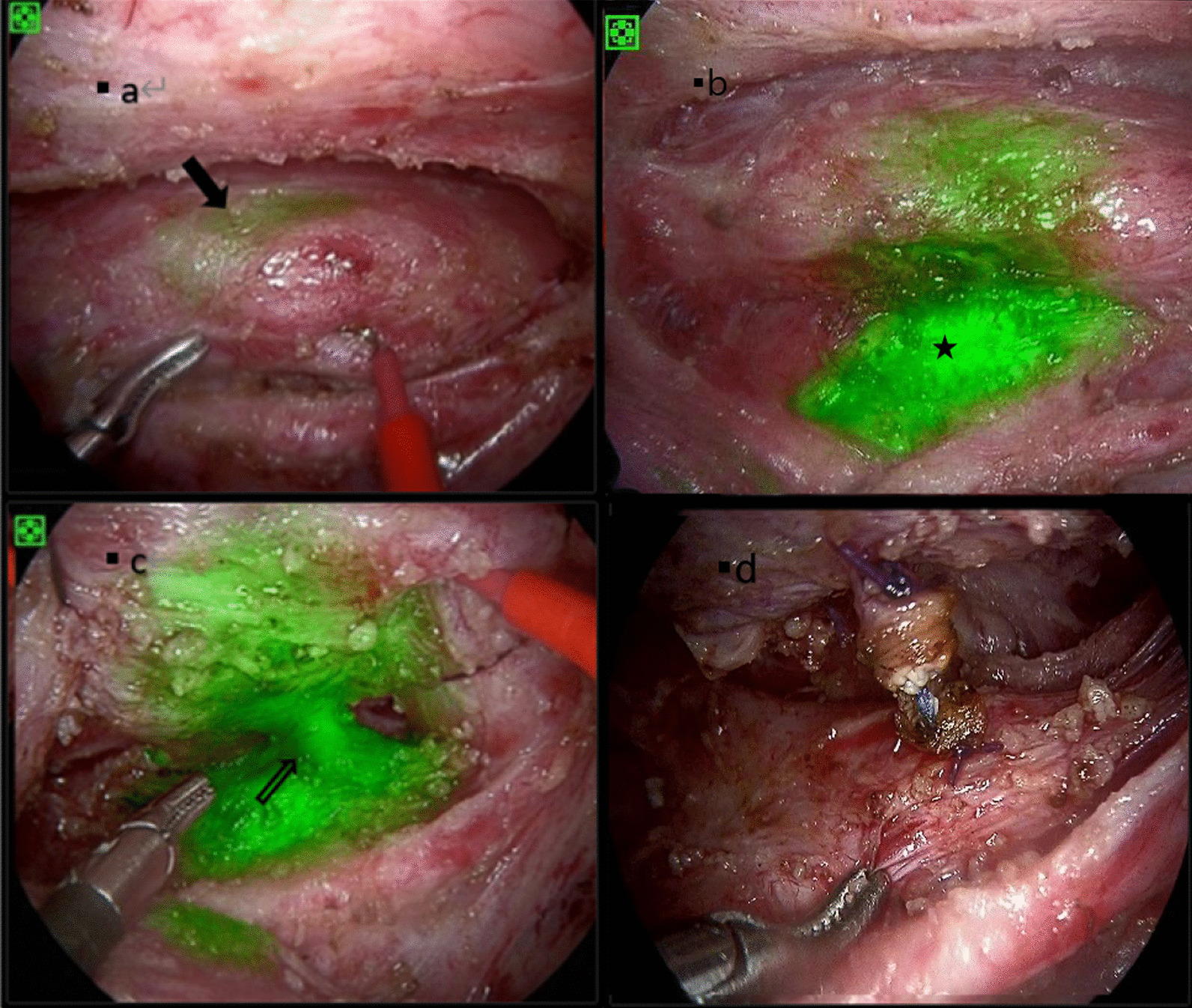
These four images showed the similar location. **a**–**c** Were with fluorescence mode turned on and d was with turned off. **a** The solid arrow was the esophagus and the fluorescence indicated the location of the fistulae; **b** separated the esophagus and trachea, we could see the bright green from trachea, five-pointed star was trachea and the esophagus lied on the upper part; **c** the hollow arrow pointed to the fistulae; **d** there was a suture inside after cutting the fistulae

The patients then underwent thoracoscopic surgery with a fluorescence imaging thoracoscope system (DPM-ENDOCAM-01). The procedure was performed via the right thorax with a three-hole approach, and the adhesions of the pleura were first separated under natural light mode. After the esophagus was exposed, the fluorescence imaging mode was turned on, and the location of the fistula was visible (Fig. 2a). The trachea was clearly visualized with bright green light by further dissecting the esophagus and exposing the trachea around the fluorescence (Fig. 2b). We avoided tissue ischemia associated with broad dissection of the esophagus and trachea during surgery by using the fluorescence imaging approach. Then, we suspended the esophagus to the chest wall with a thread to expose the fistulae (Fig. 2c). The fistulae were ligated at both ends of the esophagus and trachea and then disconnected. The silk thread was visible inside, reconfirming the accurate location of the fistulae (Fig. 2d). We removed the threads from the fistulae and stitched the fistulae with the mucous surfaces oriented toward the inside. We placed two separate layers of pedicle flaps on the esophagus and tracheal side.

##### Esophago-pulmonary fistula

**Fig. 3:**
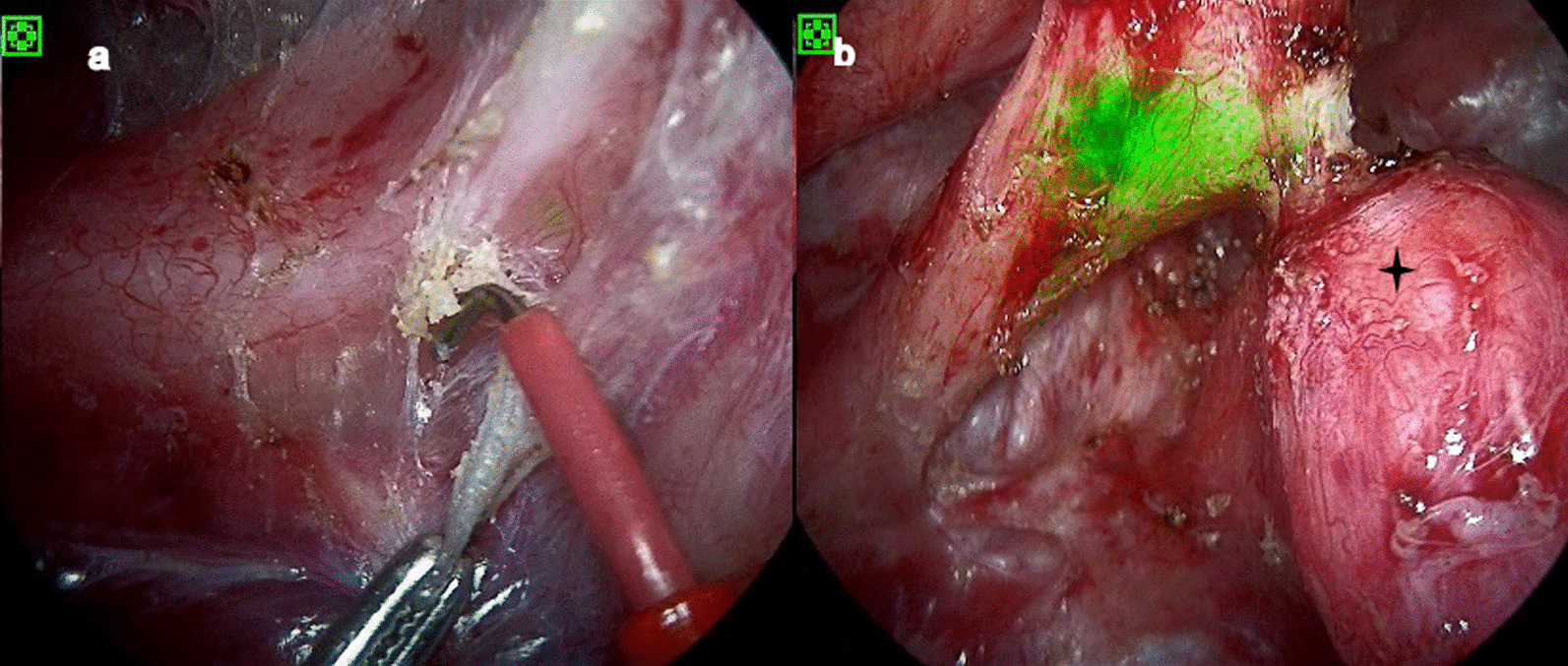
**a** At the beginning of the separating the esophagus, the light green part above the hook was location of the fistulae; **b** exposed the esophago-pulmonary fistula, black four pointed star for the pulmonary tissue

The contrast revealed overflow at the T4 level and right upper lung tissue was imaging. The bronchoscopy intraoperatively showed a depression instead of a fistula approximately 1 cm above the carina. The condition of the case didn’t allow to left the guide wire. ICG (0.5 ml) was sprayed into the depression and the opening of the right main bronchus. The operation steps were the same as those described above. The esophagus and fistula were visualized after separation of the adherent tissue from the chest wall in the fluorescence imaging mode. The fistula was located in the lung tissue surrounded by the right upper lung trachea, the right main bronchus and the main trachea (Fig. 3). Mucus secreted by alveoli could be seen after cutting the fistula. We managed with the fistula as described above.

#### Delayed esophageal closure after earlier failure of primary anastomosis

**Fig. 4:**
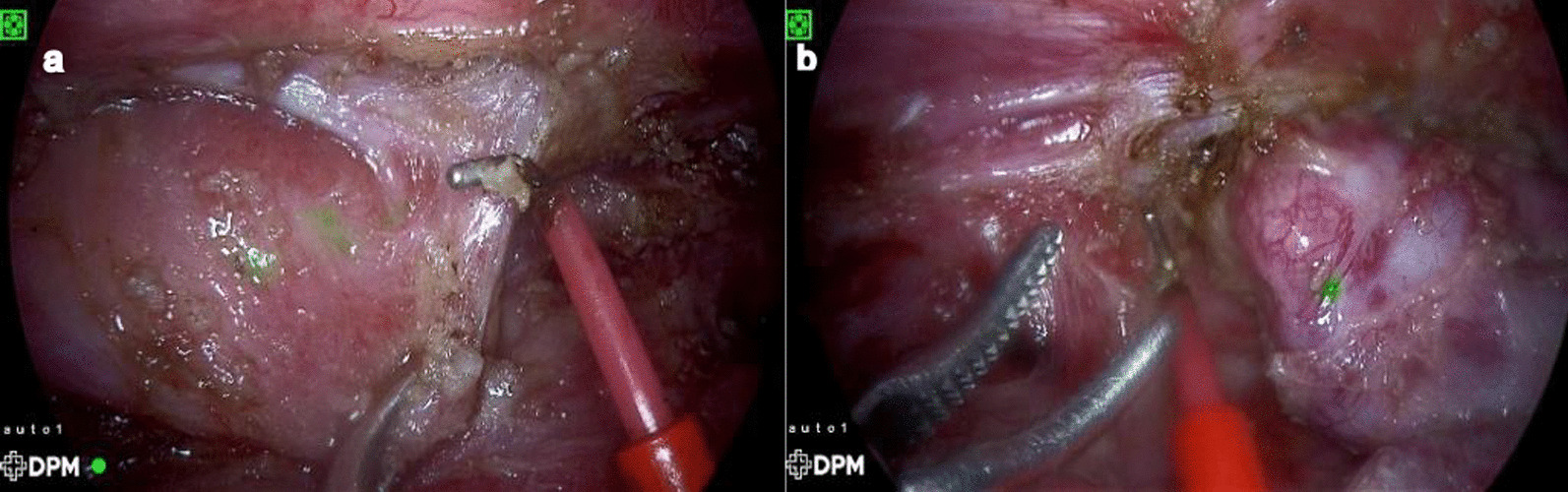
**a** The lower esophageal pouch; **b** the upper esophageal pouch; both signals were not strong

The bronchoscopy intraoperatively revealed a depression approximately 0.5 cm above the carina. However, no opening was observed. ICG (0.5 ml) was infused into the upper and lower esophageal pouches with the tube. After the separation of the adhesions from the pleura, the distal and proximal esophagus can be seen in fluorescence mode, but the visualization was weak, and the gap was 3 cm long under tension-free conditions (Fig. 4). Then, we finished the end-to-end anastomosis.

Finally, the chest tube was left in place, and the chest wall wound was stitched. The anesthesiologist drew the tracheal tube back to the primary tract beyond the fistulae and suctioned the airway to remove the sputum mixed the ICG. The patients were sent to the PICU after surgery.

## Results

Nine patients (8 males, 1 female) were included in this study. The mean age was 10 months (3–24 m). The mean weight was 7.55 kg (4.3–10.5 kg). The number of operations for each patient was 2 or 3. Classification of the primary EA was Gross-III in all patients. The comorbidities are presented in Table 1.

There were no intraoperative adverse events during surgery or side effects related to ICG spray. ICG could be detected in 100% of the cases. In the esophago-pulmonary fistula case, even though ICG was sprayed into the depression and the opening of the right main bronchus, the fistula still exhibited a strong green signal in fluorescence imaging mode. In the case of delayed esophageal closure, the signal was weaker. However, it could be enhanced by clamping and extruding the tissue slightly.

In the rTEF group, intubation with the guide wire could not be performed in 3 cases (including the EPF case). The surgeries were completed based on the indications of ICG.

The postoperative course was uneventful for 7/9 (77.8%) patients, while fistulae recurrence occurred in one patient (11.1%), and one patient required esophageal dilation because of stricture. In addition, the case of recurrent TEF was treated conservatively and temporarily.

## Discussion

Severe adhesion, fibrous tissue, and inflammation generally occur around the fistula, making rTEF repair surgery difficult. Thoracoscopic surgery provides an enlarged and clearer field. We have performed over 100 thoracoscopic repair surgeries since the first attempt in 2014, with a final cure rate of approximately 84.5% (including early cases). The success rate has been growing year after year by the increase in our thoracoscopic technical experience. The challenges of repair surgery are locating and exposing the fistula while minimizing tissue injury and ischemia. It has been proposed that a guide wire should be left in the fistula to assist in localization [9, 10] and that an endoscopic light source should be used for localization [11]. It was also reported that a fistula can be confirmed if air is seen in the trachea after complete separation of the trachea and esophagus [1]. Endoscopic light localization may interfere with respiratory support. Extra dissection of the esophagus during surgery may injure the vagal nerve and tissue more severely, even leading to lymphatic leakage [12, 13]. We also placed a guide wire prior to the operation to determine the location of the fistula as previously described by Coran [7] for double assurance. However, this may cause friction damage to the trachea and esophagus. In addition, it was difficult to insert the guide wire in some cases because of the poor esophageal condition, airway malformations and irregular fistulae. For esophageal-pulmonary fistulas, as a special type of rTEF, the thoracic infection was usually more severe, so the location could hardly be identified and precludes the use of a guide wire. We therefore attempted to improve rTEF surgery by fluorescent imaging thoracoscopy with ICG. We subsequently applied this method to delayed closure surgery. The patient was diagnosed with esophageal atresia type III with a long gap. Ligation of the tracheoesophageal fistula and gastrostomy were performed at another hospital after birth, and 3 months later, end-to-end anastomosis of the esophagus was performed and failed again. The condition of the thoracic cavity was complex and similar to that observed in patients with rTEF, and ICG helped to identify the esophageal pouch during surgery.

ICG is generated by excitation with near-infrared light (NIR) and becomes visible by the special receiver converter. The thoracoscope system for the surgery had two mode: ordinary light and fluorescence imaging mode.We can switch between two modes to help us during the procedure. We used ICG to visualize fistulae under fluorescence imaging mode during thoracoscopy. ICG is virtually nontoxic [14]. It is usually injected intravenously for the assessment of liver diseases, tumors and other conditions. The clinical application of ICG has mainly been previously reported in adults. Recently, the use of ICG in pediatric surgery has been reported to be safe and effective [6, 15]. No allergic or other adverse systemic reactions to ICG have been reported [8]. Even so, we performed the ICG skin test before surgery in case of rare allergies because the agent contained a small amount of iodine.

ICG is inexpensive and readily available, and the procedure is not time-consuming. Because of the instability of ICG in water, we suggest preparing it immediately prior to use. We obtained a perfect image by fluorescent imaging. The thickness of ICG fluorescence energy across the tissue is generally approximately 1–1.5 cm. We used a lower dose than suggested for intravenous injection, which was 0.5 ml for each operation (concentration of 1.25 mg/ml). To avoid unexpected, uncertain results, we still placed a guide wire for double assurance as the cases allowed. In surgery, fluorescence seemed strong due to the thin fistula tissue. Our fluorescence imaging system can adjust the intensity of fluorescence; thus, we could obtain a clear image. However, in the esophageal pouch, the light was weaker because of the hypertrophic tissue and scarring tissue. It could be enhanced by clamping the tissue. Thus, when the operator was uncertain, the tissue could be extruded slightly and then visualized, or we could use more dose ICG for indication. In all cases, much of the ICG was suctioned, and the rest was excreted directly via the digestive tract or through the respiratory tract as sputum. Even a small amount of drug is absorbed and fully metabolized by the liver.

There are many benefits to the application of green fluorescence indocyanine imaging demonstrated in this study. ICG shows the fistula clearly and distinctly, greatly improving the efficiency of finding the fistula intraoperatively. In addition, it is very helpful for preventing ischemia from wide dissection of the esophagus and trachea during the operation, which may also reduce the duration of the operation. Direct spray of ICG around the fistula can clearly show its location, eliminating the need for guide wire placement, which can save anesthesia time and reduce tissue damage. Moreover, ICG is beneficial for complex cases in which guide wires cannot be placed. Likewise, for in complex esophageal repair surgery, the ICG can be a good indicator.

There are also limitations to the application of ICG. There are few reports on the use of ICG in pediatric surgery. We need more cases to summarize our experience of using ICG to standardize its use and promote its broad application.

## Conclusion

In summary, the use of indocyanine green fluorescence localization-assisted thoracoscopy for revision surgery to treat complications after esophageal atresia repair is a safe and reliable method that deserves promotion.

## Data Availability

All data generated or analysed during this study are included in this published article.

## References

[1] Smithers CJ, Hamilton TE, Manfredi MA (2017). Categorization and repair of recurrent and acquired tracheoesophageal fistulae occurring after esophageal atresia repair. J Pediatr Surg.

[2] Koivusalo AI, Pakarinen MP, Lindahl HG (2015). Revisional surgery for recurrent tracheoesophageal fistula and anastomotic complications after repair of esophageal atresia in 258 infants. J Pediatr Surg.

[3] Coran AG (2013). Diagnosis and surgical management of recurrent tracheoesophageal fistulas. Dis Esophagus.

[4] Wang J, Zhang M, Pan W (2017). Management of recurrent tracheoesophageal fistula after esophageal atresia and follow-up. Dis Esophagus.

[5] Hua K, Yang S, Zhang Y (2020). Thoracoscopic surgery for recurrent tracheoesophageal fistula after esophageal atresia repair. Dis Esophagus..

[6] Lau CT, Au DM, Wong K (2019). Application of indocyanine green in pediatric surgery. Pediatr Surg Int.

[7] Shirotsuki R, Uchida H, Tanaka Y (2018). Novel thoracoscopic navigation surgery for neonatal chylothorax using indocyanine-green fluorescent lymphography. J Pediatr Surg.

[8] Esposito C, Del CF, Cerulo M (2019). Clinical application and technical standardization of indocyanine green (ICG) fluorescence imaging in pediatric minimally invasive surgery. Pediatr Surg Int.

[9] Hotta Y, Uezono S, Segawa O (2002). Precise localization of a recurrent tracheo-oesophageal fistula using retrograde guide wire placement. Paediatr Anaesth.

[10] Botham MJ, Coran AG (1986). The use of pericardium for the management of recurrent tracheoesophageal fistula. J Pediatr Surg.

[11] Bruch SW, Hirschl RB, Coran AG (2010). The diagnosis and management of recurrent tracheoesophageal fistulas. J Pediatr Surg.

[12] Nguyen T, Zainabadi K, Bui T (2006). Thoracoscopic repair of esophageal atresia and tracheoesophageal fistula: lessons learned. J Laparoendosc Adv Surg Tech A.

[13] Gutierrez RS, Guelfand M, Balbontin PV (2021). Congenital and aquired tracheoesophageal fistulas in children. Semin Pediatr Surg.

[14] Reinhart MB, Huntington CR, Blair LJ (2016). Indocyanine green: historical context, current applications, and future considerations. Surg Innov.

[15] Esposito C, Coppola V, Del CF (2020). Near-infrared fluorescence imaging using indocyanine green (ICG): emerging applications in pediatric urology. J Pediatr Urol.

